# Richter’s Hernia Unveiled: The Danger of High Pain Tolerance and Lack of Systemic Symptoms

**DOI:** 10.7759/cureus.69943

**Published:** 2024-09-22

**Authors:** Anna V Yumen, Anna Kate Wright, Henry P Moses, Nathan T Douthit

**Affiliations:** 1 General Surgery, University of South Carolina Prisma Health, Columbia, USA; 2 Surgery, Edward Via College of Osteopathic Medicine, Auburn, USA; 3 General Surgery, Prisma Health Tuomey, Sumter, USA; 4 Graduate Medical Education, East Alabama Medical Center, Opelika, USA; 5 Internal Medicine, Edward Via College of Osteopathic Medicine, Auburn, USA

**Keywords:** hernia, ischemic small bowel, stapled anastomosis, rare form of femoral hernia, open repair of femoral hernia, small-bowel obstruction

## Abstract

Hernia repairs are among the most common surgical procedures performed by general surgeons annually in the United States, defined as the abnormal protrusion of tissue and/or organs through an anatomical defect in the surrounding wall at various locations in the human body. While some hernias can remain asymptomatic and seemingly harmless, some may lead to intestinal obstruction, ischemic bowel from strangulation of blood supply, or septic shock if not diagnosed and addressed within a short period of time. This case report is about an elderly woman who presented with a Richter’s. A Richter’s hernia is an atypical type of strangulation where only a portion of the bowel, the antimesenteric border, is trapped within the anatomical defect. Given the limited extent of entrapped bowel, numerous different presentations can be seen, including but not limited to signs of obstruction without signs of ischemia or, in some instances, lack of either sign of obstruction or ischemia. Within this report, we will discuss the need for high clinical suspicion for Richter’s hernias when evaluating strangulated hernias without systemic signs of sepsis.

## Introduction

A Richter’s hernia is a partial protrusion or strangulation of the bowel’s antimesenteric border through a defect in the abdominal wall. It is a rare occurrence, making up only 10% of strangulated groin hernias [1]. The first known case was identified by Fabricius Hildanus in 1606 and was given a definition in 1785 by August Gottlieb Richter [1]. With a Richter’s hernia, only a portion of the loop of the bowel protrudes through the small anatomical defect [2]. The most susceptible portion of the bowel to become trapped is the distal ileum [2]. Richter’s hernias are encompassed under “groin hernias” as they are most notably associated with femoral hernias followed by inguinal hernias [2,3]. Given the more common presentation in femoral hernias, they are often identified within the elderly female population [4]. The danger that arises from this particular hernia is its ability to evade detection for a prolonged period of time. Due to the limited portion of the bowel that becomes trapped within the anatomical defect, there is often a lack of obstructive symptoms for a prolonged period of time. There may be no systemic signs of ischemia as the incarcerated loop of bowel and blood supply is trapped within the defect, and no markers of ischemia are released into the systemic vasculature. With the indolent course of presentation and identification, these cases become far more urgent and can be associated with more dangerous complications, including bowel perforations, gangrenous intestines, wound complications, skin necrosis, and enterocutaneous fistulas [2,5,6]. Throughout the past two decades, there has been a noted increased prevalence of diagnosed Richter’s hernias [5]. This increase has been attributed to the increased nature of minimally invasive surgery. Through minimally invasive surgery, smaller defects seem to be produced, allowing for only a portion of the intestine to be trapped and strangulated. Within this report, we will discuss a presentation of a Richter’s hernia and the underlying need for a high index of suspicion to promptly diagnose and treat a patient with a Richter’s hernia.

## Case presentation

**Figure 1:**
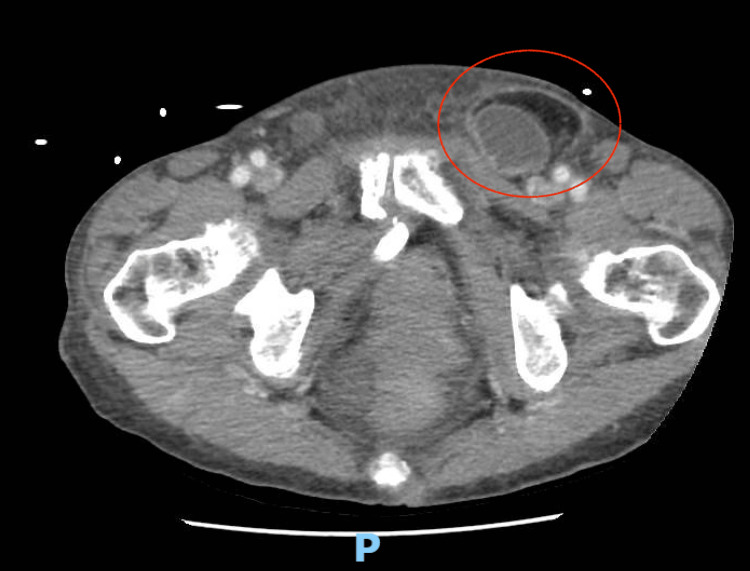
CT image depicting left lower abdominal mass containing bowel.

An 87-year-old female presented to the emergency department with a chief complaint of abdominal pain, nausea, and vomiting. She claimed that over the course of a week, she had noticed significant abdominal distension, as well as a persistent firm left inguinal bulge. The patient reported that within the past two days, she had developed the associated nausea, vomiting, and oral intolerance, which led her to seek further medical evaluation. She denied recent bowel movements or passing of flatus. The patient reported overall good physical health with no previous medical diagnoses or daily medications. She reported having a previous abdominal surgery, a nephrectomy, when she served as a transplant donor for a friend back in Japan. On presentation, the patient did not display any signs of systemic illness, as she was afebrile, hemodynamically stable, and non-tachycardic. On exam, the patient appeared non-toxic and in no acute distress. The patient’s abdomen was protuberant yet also diffusely soft and minimally tender, absent of any peritoneal signs. A firm, tender left inguinal bulge was noted that had no overlying skin changes and was non-reducible. A CT scan of the abdomen and pelvis was obtained by the emergency department, which showed findings consistent with a small bowel obstruction with a transition point at the left femoral hernia (Figure 1). The hernia contained loops of small bowel with surrounding edema and fluid concerning ischemia. Of note, the patient did not display signs of infection on lab work, as she had a normal white blood cell count of 9.5 (reference range: 4.5 to 11.0 × 10^9^/L) and lactate within normal limits at 1.1 (reference range: >2mmol/L).

After evaluation and discussion with the patient regarding the concern of ischemic bowel from the femoral hernia defect, a nasogastric tube was placed and she was urgently taken to the operating room. Given her history of intra-abdominal surgery and a concern for intra-abdominal adhesions, the decision was made to proceed with an open inguinal repair. An incision was made in the left inguinal area between the ASIS and pubic tubercle. The incision progressed through Scarpa’s fascia down through the external oblique aponeurosis. At this time, we did not encounter an inguinal hernia; however, given our concern for a femoral hernia on exam and imaging, we opened the inguinal floor and transversalis fascia. Immediately upon entry, a hernia sac was encountered protruding through the femoral canal. The sac was noted to be dark in color hue, consistent with concern of intestinal ischemia. The hernia sac was opened and ischemic omentum and bowel were found. Given the small femoral defect, we were unable to withdraw any remaining viable bowel. Despite our attempt at creating a larger defect to excise further bowel, we did not feel we had adequate bowel length to allow us to perform a bowel resection through an isolated inguinal incision. At this point, we converted to a minilaparotomy to complete the bowel resection.

**Figure 2:**
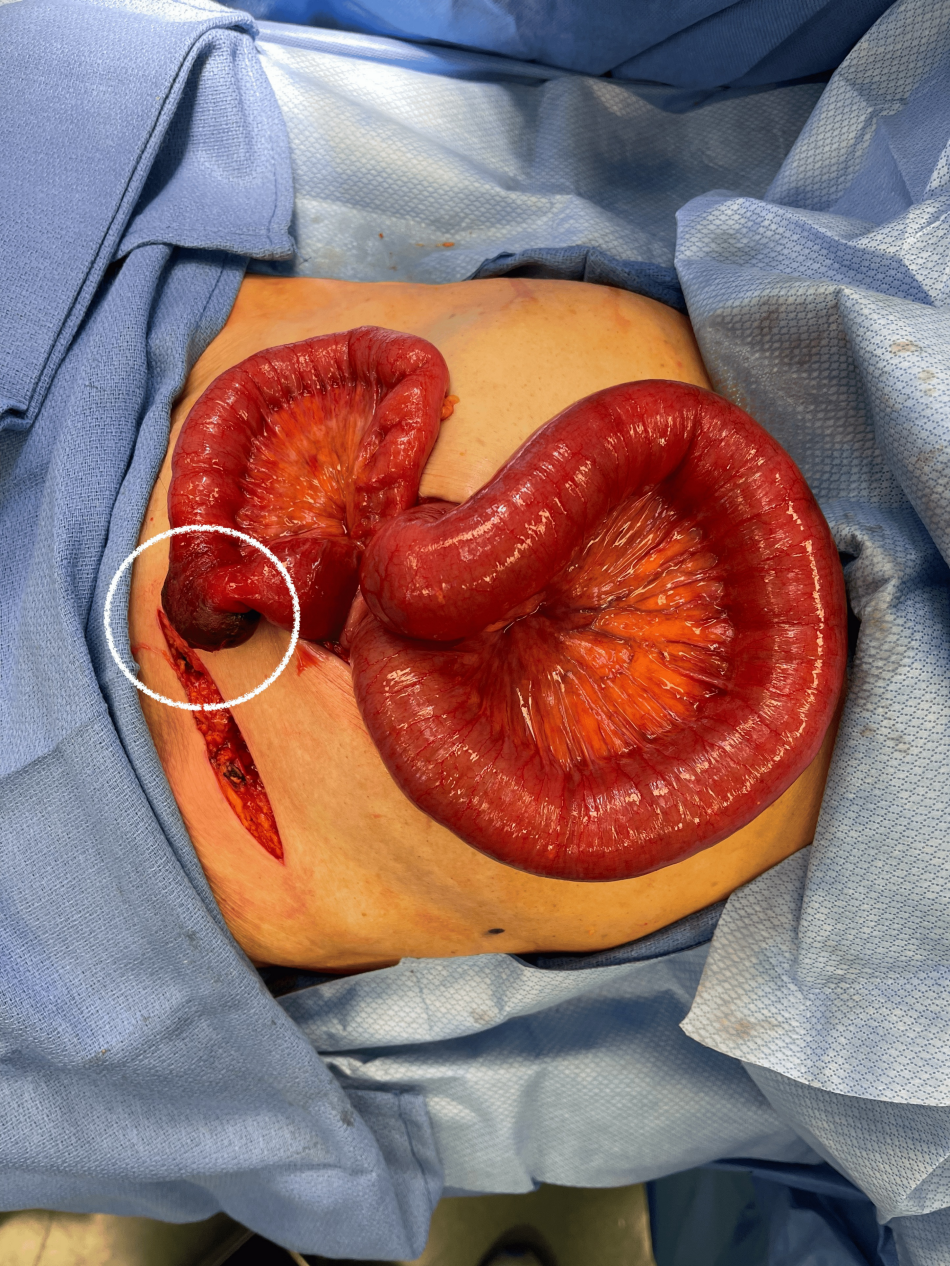
A large segment of the bowel lies outside the abdominal cavity. The stark contrast between healthy tissue and the ischemic segment can be noted.

**Figure 3:**
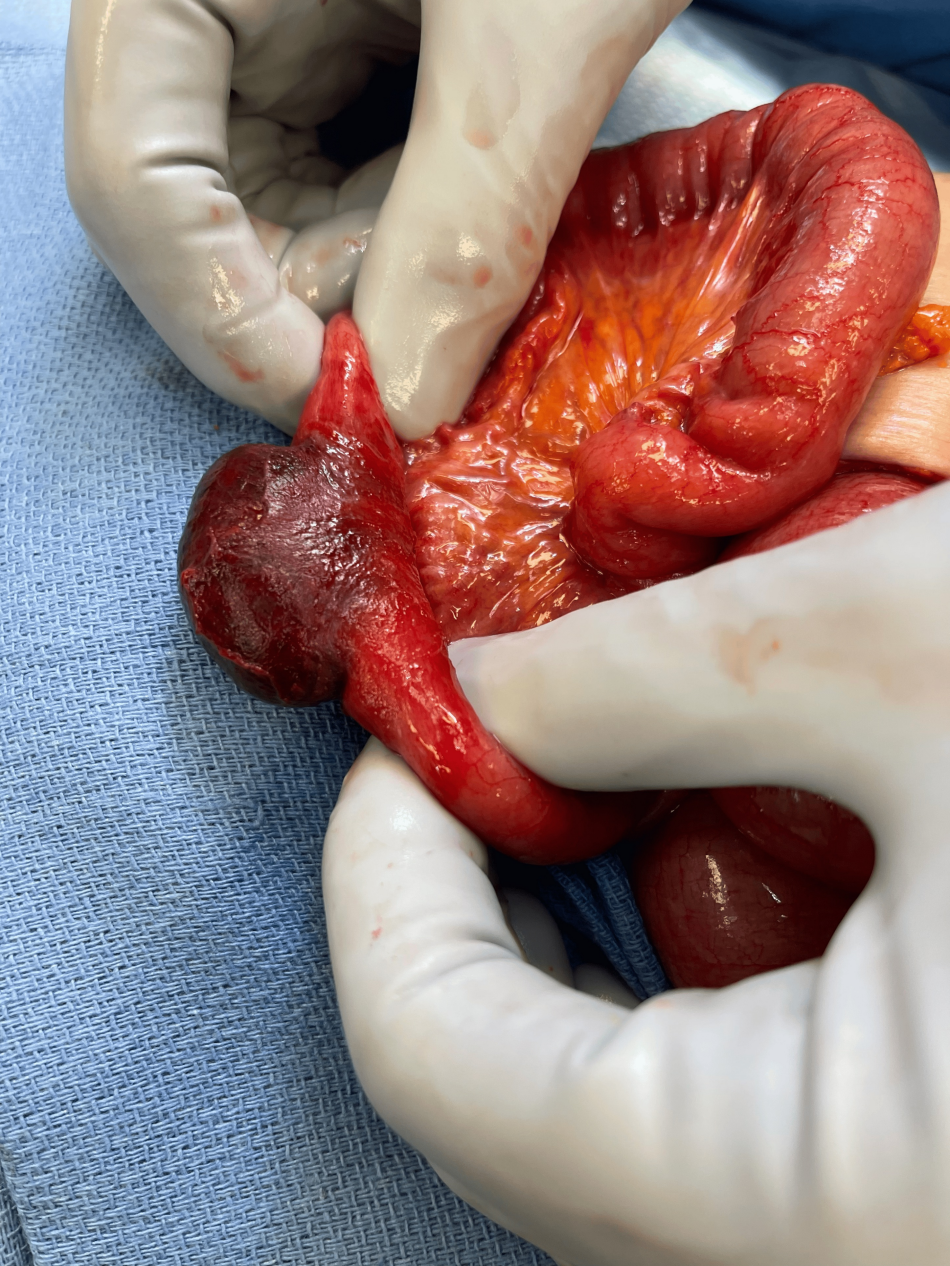
A closer look at the Richter’s hernia with its ischemic outer segment and viable inner segment.

A midline incision was made through the fascia and entered the abdominal cavity. Upon entry, a few abdominal adhesions were encountered and lysed. In addition, dilated proximal loops of small bowel were seen, consistent with a small bowel obstruction. Through the inguinal incision, we reduced the ischemic bowel back into the abdominal cavity, allowing us to eviscerate the small bowel in its entirety. We ran the small bowel from the ligament of Treitz to Treves and, at this time, noted that the femoral hernia was also to be classified as a Richter’s hernia, as only the antimesenteric border of the bowel was ischemic (Figures 2, 3).

With no signs of contamination from intestinal perforation or spillage, the decision was made to proceed with our femoral hernia repair with mesh placement prior to our small bowel resection and re-anastomosis. We proceeded to perform a modified Lichtenstein hernia repair, also known as a “Plug and Patch Mesh Repair.” The remaining hernia sac was transected, given that the patient was a female, and then copiously irrigated prior to proceeding with our hernia repair. A mesh plug was placed and fixed into the internal ring prior to placing a separate sheet of mesh to re-create the inguinal floor. The mesh was fixed to the pubic tubercle and the shelving ledge of the inguinal ligament. The mesh continued beyond the internal ring approximately 3 cm, allowing the re-creation of the internal ring. We proceeded to re-approximate the external fascia, Scarpa’s, and skin prior to returning to the abdomen and bowel resection.

Once our femoral hernia repair was completed, we re-assessed the small bowel in its entirety, with the only pathologic finding being our segment of known ischemic bowel. Using two linear 75 cm blue staple loads and a Ligasure™ vessel sealing device (Stryker), approximately 10 cm of bowel was resected, encompassing the portion of the ischemic bowel. We then completed the bowel anastomosis in a stapled isoperistaltic side-to-side functional end-to-end fashion. Two stay sutures were placed in accordance with the staple lines as well as two 2-0 absorbable anchoring sutures that were used to align the pieces of bowel, taking care to align the antimesenteric borders for the planned enterotomies. Enterotomies were then established on each segment of the bowel at the antimesenteric borders on the ends of the proximal and distal segments. Next, a cutting linear stapler was carefully inserted into each segment of the bowel, and a common channel was made. The channel was then closed with a blue linear stapler load. There was no contamination, and the anastomosis was palpated without concern. The mesenteric defect was closed. The bowel was returned to the abdominal cavity. We proceeded to irrigate the cavity and then closed the abdomen in standard fashion.

Post-operatively, the patient had a return of bowel function and was able to progress with her diet. Her recovery was uneventful and she was discharged home on post-operative day 4. 

## Discussion

While any hernia can pose significant morbidity and mortality risks, due to the nature of a Richter’s hernia and the likelihood of indolent symptoms, often its pathologic state is much more advanced at the time of presentation despite its benign appearance. Patients oftentimes do not seek medical attention as, due to the anatomic nature of Richter’s hernia, they continue to have functioning bowel and minimal, if any, systemic signs of illness [4]. Patients have been noted to seek medical attention upon presentation of more severe signs, such as gangrenous bowel, skin necrosis, enterocutaneous fistula, or frank perforation [2,4,5]. Recent research has been conducted and shows a potential benefit to using the De Ritis ratio, aspartate aminotransferase (AST) to alanine aminotransferase (ALT) ratio (AST:ALT), to detect bowel necrosis and incarcerated hernias. A study was conducted using the De Ritis ratio as an inflammatory marker for those with known hernias and discovered that there was a decreased risk of mortality for those who saw an increased AST:ALT and proceeded with surgical intervention [7]. However, ALT and AST are often indicators of other pathology associated with hepatic disease and may not be specific enough to possible bowel necrosis. Ultimately, it is up to the clinician to have a high index of suspicion to evaluate for underlying strangulation. While in our case there was a clear inability to reduce the patient’s hernia, even if the hernia is reducible with manipulation, there is a high risk of morbidity and mortality by releasing the ischemic segment of the bowel back into the abdominal cavity and allowing the systemic effects. The timing upon presentation and adjuncts of lab work and imaging can assist with the diagnosis of ischemic bowel; however, the only true evaluation is visual inspection within the operating room. While immediate surgical exploration is the standard approach, there are many different approaches in how to assess the bowel. In our case, given the history of prior intra-abdominal surgeries and pre-operative identification of a femoral hernia, we felt an open repair was best. With any approach, the most important factor is to carefully assess the viability of the bowel once it is freed. Any suspicion of compromised tissue warrants resection and anastomosis [4]. It is equally as important to ensure appropriate hernia repair in an effort to prevent another future incident through the same abdominal defect [1,4]. While early identification and repair help limit complications, complications such as enterocutaneous and colocutaneous fistulas may still arise [4]. These circumstances often require a more extensive surgical approach and prolonged recovery [4]. Overall, a Richter’s hernia is a rare diagnosis; however, one must be diligent to closely evaluate patients with incarcerated hernias despite their overall non-toxic appearance, as a masked Richter’s hernia may be present.

## Conclusions

As with any incarcerated hernia, medical professionals must work diligently to assess for and identify compromised bowel. This specific case report highlights a patient with a Richter’s hernia who presented in a delayed fashion due to the indolent course of symptoms that are often seen in these specific hernias. As with our patient, due to the delayed presentation, patients often present with advanced progression of the disease, and ischemic bowel is a common encounter. This case emphasized how Richter’s hernias present in a delayed fashion with serious surgical needs necessitating prompt surgical intervention and treatment. Our patient highlights the need for more education and exposure to Richter’s hernias to prevent serious complications that can arise within this population.
